# The intron 4c allele of the NOS3 gene is associated with ischemic stroke in African Americans

**DOI:** 10.1186/1471-2350-8-76

**Published:** 2007-12-10

**Authors:** RP Grewal, AVC Dutra, Yi C Liao, Ss H Juo, NIH Papamitsakis

**Affiliations:** 1 New Jersey Neuroscience Institute at JFK Medical Center, 65 James Street, Edison, NJ 08818, USA; 2 Graduate Institute of Medicine, Kaohsiung Medical University, Kaohsiung 800, Taiwan; 3 Department of Neurology, Kaohsiung Medical University Hospital, Kaohsiung 800, Taiwan; 4 Department of Neurology, Medical University of South Carolina, 96 Jonathan Lucas Street, Charleston, SC, 29425, USA

## Abstract

**Background:**

Ischemic stroke is the most common cause of disability in North America and in addition to the generally accepted risk factors, there is increasing evidence for the potential pathophysiological role of genes. One of these genes, the endothelial nitric oxide synthase gene (*NOS3*) has been reported as a genetic risk factor for ischemic stroke. To independently confirm and extend the results of these previous reports, we investigated this gene as a risk factor for stroke in an ethnically diverse study population.

**Methods:**

Using the TOAST classification, we characterized and studied 377 patients with ischemic stroke. We genotyped two common variants in the *NOS3 *gene, the intron 4 insertion/deletion and an exonic single nucleotide polymorphism (SNP), G894T, in these patients and compared them with 502 controls. Chi-square or Fisher's exact tests were used to examine allele effects on stroke and stroke subtypes. Logistic regression analysis was used to adjust for confounding covariate effects.

**Results:**

All genotypes are in Hardy-Weinberg equilibrium except for intron 4c, which is overrepresented in ischemic stroke patients. In pooled analysis of all patients, intron 4c, but not intron 4a, intron 4b or G894T alleles are associated with stroke (p < 0.01). In subgroup analysis by race, the intron 4c allele is most strongly associated with large artery ischemic stroke in African Americans (p < 0.01).

**Conclusion:**

We are unable to confirm previous reports of an association of the intron 4a or the G894T alleles with ischemic stroke. However, although limited by a relatively small sample size, our study suggests a potentially important role of the intron 4c allele as a genetic marker of ischemic stroke in African Americans.

## Background

Ischemic stroke is the leading cause of disability in North America and the third most common cause of death [1,2]. Since age is a major risk factor for stroke, as the population ages, the burden from this disease will continue to increase. In addition to the commonly accepted risk factors, there is increasing evidence for the role of genes in the pathophysiology of cerebrovascular disease [3]. The endothelial nitric oxide synthase gene, (*NOS3*), has been the focus of recent investigations as a potential genetic risk factor in the development of ischemic stroke [4-6].

The *NOS3 *gene encodes an isozyme of nitric oxide synthase that, in turn, catalyzes the generation of nitric oxide (NO). The release of NO by endothelial cells mediates vascular relaxation in response to vasoactive substances and shear stress. In addition, it mediates antiproliferative and antithrombotic action by inhibiting vascular smooth muscle cell proliferation, monocyte adhesion, platelet aggregation, and thrombosis [7]. This participation of the *NOS3*gene in the physiology of the vasculature makes it a biologically plausible candidate for study as a susceptibility gene in ischemic stroke.

A genetic contribution of *NOS3 *to plasma levels of NO has been reported [8]. Furthermore, two potentially biologically relevant genetic polymorphisms have been studied. The first is an intron 4 insertion/deletion or the variable number tandem repeat (VNTR) polymorphism and the second is an exonic single nucleotide polymorphism (SNP), G894T (Glu298Asp) [6]. The intron 4 VNTR consists of repeats of a 27-bp consensus sequence, which may be repeated 4 times (intron 4a allele), 5 times (intron 4b allele) or 6 times (intron 4c allele)[4]. Although the results are conflicting, there are reports implicating variants in both these polymorphisms in the development of ischemic stroke [4-6]. To confirm and extend the results of these studies, we investigated both polymorphisms as susceptibility loci for ischemic stroke in an ethnically diverse population of patients.

## Methods

### Human Subjects

We studied 377 patients admitted to the stroke unit at New Jersey Neuroscience Institute/John F. Kennedy Hospital, Edison, New Jersey. All patients were confirmed to have suffered an ischemic stroke with imaging data, either by cerebral MRI or CT imaging. In addition, 502 controls patients with no clinical or radiological evidence of cerebrovascular disease were recruited from local family practice offices and from patients evaluated at the New Jersey Neuroscience Institute, Edison, New Jersey. All patients were entered into a stroke database recording the demographic and clinical data (Table 1). Strokes were categorized according to the Trial of ORG 10172 in Acute Stroke Treatment (TOAST) [9] and Oxfordshire criteria [10]. Race was defined by self-report, however, all patients and controls were under the care of physicians who provided independent reporting. Any discrepancies were resolved by direct communication with the patient or family. Blood samples were obtained by policies and procedures approved by the local Institutional Review Board.

**Table 1:** The clinical parameters of stroke and control groups.

	Control group (n = 502)	Case group (n = 377)
Age (yrs)	65.32 ± 14.83	69.92 ± 13.52 ^†^
Gender (male, %)	41.2%	48.3%*
Hypertension (%)	55.1%	76.0%^†^
Diabetes (%)	24.6%	37.4%^†^
Ex-smoker/Current smoker (%)	8.6%/17.7%	8.7%/17.9%
Race (Caucasian/African American/Asian-Indian/Hispanic/Asian-oriental/Others) (%)		
	81.7%/8.4%/3.0%/3.8%/0.8%/2.4%)	68.4%/11.9%/5.8%/6.9%/4.0%/2.9%)^†^
TOAST classification		
Cardioembolism	NA	24.1%
Large artery atherosclerosis	NA	31.8%
Small vessel occlusion	NA	24.1%
Others / unknown	NA	19.9%
Glu298Asp allele distribution G/T (2n)	482/198	460/194
Intron 4 VNTR allele distribution a/b/c (2n)	164/830/4	127/590/15^†^
Caucasian	126/688/2	87/410/3
African American	23/57/2	20/57/11*
Asian-Indian	3/27/0	7/36/1
Hispanic	5/33/0	9/41/0
Asian-Oriental	2/6/0	1/27/0
Others	5/19/0	3/19/0

*p < 0.05, ^†^p < 0.01, the clinical data were expressed as mean ± SD where appropriate.

NA = not applicable

### Genetic Materials and Methods

DNA was extracted from a blood sample by standard methods (Gentra Systems, Minneapolis, MN). Genotyping was performed by polymerase chain reaction (PCR) in which all reactions were performed in a total volume of 50 μl, using 2.5 U of Ampli-Taq (Promega); 600 μM of a dNTP mix and 40 ng of DNA template. The intron 4 polymorphism genotyping was performed as previously described [4]. The allele sizes, a-393 bp, b-420 bp and c-447 bp were determined by gel electrophoresis. The G894T polymorphism was genotyped by PCR employed previously published conditions and primers [6]. The products were digested to completion with Mbo1 (New England Biolabs) and visualized in a 6% polyacrylamide gel. For both polymorphisms, the reliability of the genotyping was confirmed by direct sequencing of more than 20 randomly chosen samples of each genotype.

### Statistical Analysis

The chi-square or Fisher's exact tests were used to examine the allelic effect on stroke and stroke subtypes. Logistic regression analysis was employed to adjust for the confounding effects of covariates. Covariates in the regression model included age, gender, diabetes and hypertension. Smoking status (ex- and current smoker vs. non-smoker) was not significantly related to disease and was excluded from the logistic regression analysis.

## Results

The clinical and genetic data are presented in Table 1. Genotyping data for the Glu298Asp SNP was obtained on 340/502 controls and 327/377 cases and for the intron 4 VNTR polymorphism, on 499/502 controls and 366/377 cases. On some of the samples, only one polymorphism was genotyped. There were no genotyping failures.

The Glu298Asp SNP genotypes are in Hardy-Weinberg equilibrium (HWE). Analysis of the intron 4 VNTR polymorphism shows that the control group is in HWE, but not the cases due to the over represented intron 4c allele in African American patients.

The common allele G of the G894T polymorphism has a similar frequency between control and stroke group (71% vs. 70%). Neither the G nor T allele of the Glu298Asp polymorphism is associated with higher stroke risk in either the pooled data or in the subgroup analysis by stroke subtype or race (Table 2)

**Table 2:** Allele frequencies of candidate genes among each stroke subtype.

Gene	Races	Allele	Controls 2n (%)	All Cases 2n (%)	TOAST classification, 2n (%)			
					
					Large artery atherosclerosis	Small vessel occlusion	Cardio- embolism	Others
Intron 4 VNTR	All	b	830 (83.2)	590 (80.6)	186 (78.2)	142 (80.7)	145 (83.3)	117 (81.3)
		a	164 (16.4)	127 (17.3)	40 (16.8)	34 (19.3)	27 (15.5)	26 (18.1)
		c	4 (0.4)	**15 (2.0) **^†^	**12 (5.0) **^†^	0 (0.0)	2 (1.1)	1 (0.7)
	Caucasian	b	688 (84.3)	410 (82.0)	129 (81.6)	100 (80.6)	112 (82.4)	69 (84.1)
		a	126 (15.4)	87 (17.4)	26 (16.5)	24 (19.4)	24 (17.6)	13 (15.9)
		c	2 (0.2)	3 (0.6)	3 (1.9) *	0 (0.0)	0 (0.0)	0 (0.0)
	African American	b	57 (69.5)	57 (64.8)	13 (46.4)	12 (75.0)	16 (80.0)	16 (66.7)
		a	23 (28.0)	20 (22.7)	7 (25.0)	4 (25.0)	2 (10.0)	7 (29.2)
		c	2 (2.4)	11 (12.5) *	**8 (28.6) **^†^	0 (0.0)	2 (10.0)	1 (4.2)
Glu298Asp	All	G	482 (70.9)	460 (70.3)	151 (71.2)	111 (72.1)	99 (64.3)	99 (73.9)
		T	198 (29.1)	194 (29.7)	61 (28.8)	43 (27.9)	55 (35.7)	35 (26.1)
	Caucasian	G	392 (68.8)	303 (67.6)	98 (67.1)	74 (69.8)	74 (61.7)	57 (75.0)
		T	178 (31.2)	145 (32.4)	48 (32.9)	32 (30.2)	46 (38.3)	19 (25.0)
	African American	G	44 (84.6)	59 (73.8)	20 (83.3)	13 (81.2)	10 (62.5)	16 (66.7)
		T	8 (15.4)	21 (26.2)	4 (16.7)	3 (18.8)	6 (37.5)	8 (33.3)

*p < 0.05, ^†^p < 0.01, 2n refers to the allele count.

The common allele of the intron 4 VNTR polymorphism, the "b" allele has a frequency of 81% in cases versus 83% in control subjects. If the intron 4b allele is used as a reference, the distribution of the intron 4c allele is significantly different between stroke patients and healthy controls (2% in cases vs. 0.4% in controls, p = 0.001). Furthermore, the frequency of this allele varies across different ethnic groups with the highest prevalence (7.6%) in African- American patients. Overall, the intron 4c allele increases the risk of stroke 5.3 fold compared to the b allele (OR = 5.3, p = 0.001, 95% C.I. 1.9–18.6). Stroke subtype analysis using the TOAST classification system shows that the c allele effect exclusively appears in the large artery subtype.

The genetic effect may be confounded by race if the data is pooled from all ethnic groups and analyzed as a single population. Therefore, we performed an race-specific analysis which shows that African Americans with stroke have a significantly higher prevalence of intron 4c allele than controls (12.5% vs. 2.4%, p = 0.018). The c allele increases the risk for stroke by 5.5 fold compared with the b allele. This increased risk appears to be limited to those who suffered a stroke secondary to large artery atherothrombosis but not other subtypes of stroke. In Caucasians, the intron 4c allele is also more prevalent in patients with the large artery subtype (1.9% vs. 0.2%, Fisher p = 0.032). However, given that this allele is extremely rare in Caucasians, these results should be interpreted cautiously.

In Caucasians and African Americans, logistic regression analysis was performed with adjustment for age, gender, hypertension, and diabetes as covariates (Table 3). This analysis showed the adjusted odds ratio of the c allele was similar to the crude one. In other ethnic subgroups, the c allele is too rare for any meaningful analysis.

**Table 3:** Crude and adjusted odd ratios for stroke risk factors and gene in logistic regression analysis.

Risk factors		Overall	Caucasian	African American
Crude O.R. of intron 4 VNTR (cc+ac+bc vs. aa+ab+bb)		3.84 (1.21 – 12.14), p = 0.02	1.64 (0.23 – 11.70), p = 0.62	4.33 (0.86 – 21.77), p = 0.08
Adjusted O.R.	c allele of intron 4 VNTR (cc+ac+bc vs. aa+ab+bb)	3.54 (1.08 – 11.60), p = 0.04	1.48 (0.20 – 10.96), p = 0.70	5.06 (0.81 – 31.53), p = 0.08
	Age	1.02 (1.01 – 1.03), p < 0.01	1.03 (1.01 – 1.04), p < 0.01	1.03 (0.99 – 1.07), p = 0.14
	Gender	1.45 (1.09 – 1.93), p < 0.05	1.46 (1.05 – 2.04), p < 0.05	1.13 (0.38 – 3.42), p = 0.82
	Hypertension	1.99 (1.44 – 2.75), p < 0.01	1.68 (1.15 – 2.45), p < 0.01	2.75 (0.88 – 8.64), p = 0.09
	Diabetes	1.56 (1.14 – 2.12), p < 0.01	1.38 (0.96 – 1.99), p = 0.08	2.34 (0.81 – 6.75), p = 0.12

Crude O.R. of Glu298Asp (TT+GT vs. GG)		1.10 (0.81 – 1.49), p = 0.55	1.11(0.78 – 1.58), p = 0.56	2.25 (0.80 – 6.35), p = 0.13
Adjusted O.R.	T allele of Glu298Asp (TT + GT vs. GG)	1.11 (0.81 – 1.53), p = 0.51	1.12 (0.78 – 1.61), p = 0.55	2.84 (0.79 – 10.14), p = 0.11
	Age	1.01 (0.99 – 1.02), p = 0.06	1.02 (1.01 – 1.04), p < 0.05	1.04 (0.99 – 1.08), p = 0.09
	Gender	1.39 (1.01 – 1.91), p = 0.05	1.44 (0.99 – 2.08), p = 0.05	1.10 (0.34 – 3.55), p = 0.87
	Hypertension	1.96 (1.37 – 2.81), p < 0.01	1.65 (1.08 – 2.50), p < 0.05	2.72 (0.72 – 10.28), p = 0.14
	Diabetes	1.54 (1.08 – 2.18), p < 0.05	1.41(0.93 – 2.13), p = 0.10	2.15 (0.59 – 7.90), p = 0.25

The 95% confidence interval of odds ratio (O.R.) was expressed in parentheses.

## Discussion

There have been previous studies of the G894T polymorphism in stroke. In French patients, an association between the "G" allele of the G894T polymorphism was found in patients with ischemic infarcts, especially lacunar strokes [6]. This was not supported by investigations of either Scottish or English patients [5,11]. Similar to these latter studies, our results do not support an association between the Glu298Asp polymorphism and stroke. However, given the relatively small sample size of lacunar strokes in our study population, our negative result is must be interpreted with caution.

In a study of 364 Chinese patients and 516 controls, the intron 4a allele was reported as an independent risk factor for developing ischemic stroke [4]. In English patients, this same allele showed a protective affect in the development of small vessel disease [6]. In contrast to the study of Chinese patients, our analysis shows no association of the 4a allele with stroke. Interestingly, our investigation does show an association of the intron 4c allele and stroke, especially in African Americans.

The frequency of the intron 4c allele depends upon the ethnic make up of the population. In a study of 1,038 German patients with coronary artery disease and/or hypertension, the c allele was rare, present in only 5 individuals (0.005%) [12]. In the Chinese, this allele appears to be even less common and was not observed in any of the 880 ischemic stroke patients/controls genotyped [4]. In an investigation of the relationship between asthma and the intron 4 polymorphisms, it was noted that the frequency of the c allele was 3/390 (0.8%) in Caucasians versus 27/510 (5.3%) in African Americans [13]. Our analysis of the epidemiology of the intron 4 alleles is comparable to the data obtained in these studies and indicates a higher frequency of the c allele among African-Americans (Tables 1 and 2).

There has been one report studying the *NOS3 *gene in a biracial population of Caucasians and African-Americans with ischemic stroke [14]. In that investigation, the *NOS3 *gene was analyzed by genotyping 3 polymorphisms in the promoter in addition to the intron 4 and G894T alleles. The study was conducted in young woman with ischemic stroke with a sample size of 110 cases (46% African American) compared to 206 controls (38% African American). It was reported that in African American women, two SNPs in the *NOS3 *promoter but not the intron 4 or G894T alleles were associated with ischemic stroke. However, further analysis of their data using the most common b allele as the reference shows that the OR for the c allele in African Americans was 4.76 (p = 0.041). This is similar to our result and provides further support for the potential importance of this polymorphism in ischemic stroke among African Americans.

Although our results regarding intron 4c are suggestive, they involve small sample sizes that in turn limits the statistical analysis used in power calculations (Table 3). Further study with large samples sizes of African-American patients with ischemic stroke will needed to confirm and extend these results.

## Conclusion

In contrast to some previous reports, our study fails to confirm any association of the G894T or intron 4a polymorphisms with ischemic stroke. However, the results of all these studies may be influenced by ethnic differences in allele frequencies and genetic susceptibility. The importance of such ethnic differences in these kinds of studies is illustrated by our analysis showing an association of the intron 4c allele with ischemic stroke in African-Americans. Further study of the intron 4c allele in ischemic stroke is warranted.

## Competing interests

The author(s) declare that they have no competing interests.

## Authors' contributions

AVD collected patient data, performed the genotyping and prepared a draft of the manuscript. YCL and SSHJ performed the genetic statistical analysis and revised the manuscript. NIHP reviewed the patient data and contributed to drafts of the manuscript. RPG conceived of the idea, developed the study design and was responsible for overall supervision of all aspects of this research project. All authors read and approved of the final manuscript.

## Pre-publication history

The pre-publication history for this paper can be accessed here:


